# Supplementary material for: Single-cell transcriptomics of peripheral blood in the aging mouse
Source: Aging (Albany NY). 2023 Jan 6;15(1):6–20. doi: 10.18632/aging.204471 (PMC9876630; doi:10.18632/aging.204471)
Supplement: Supplementary Figures [file aging-15-204471-s001.pdf]

SUPPLEMENTARY FIGURES

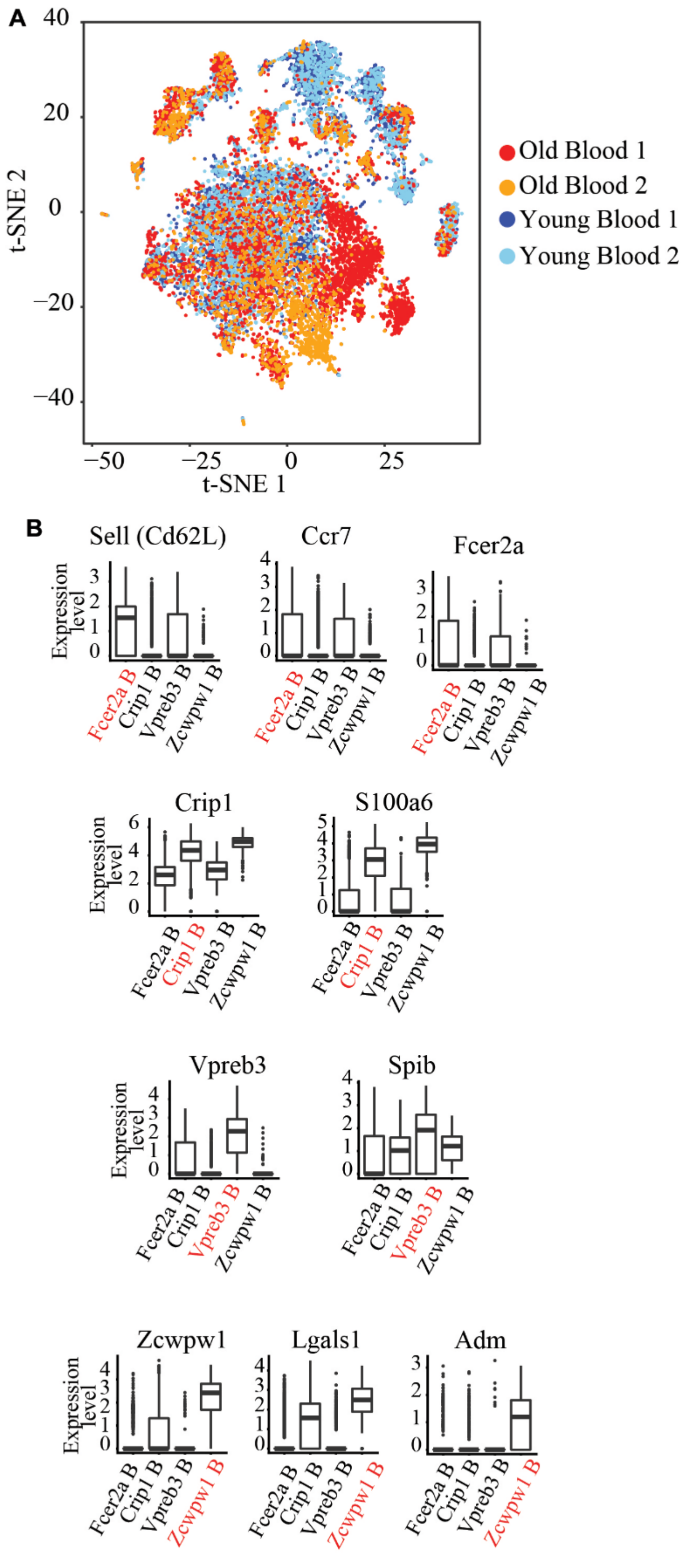

Supplementary Figure 1. (**A**) t-SNE visualization of young and old blood replicates. (**B**) Boxplots showing the expression levels of the respective marker genes in each B cell cluster. The cluster that the B cells are identified in was indicated in red.

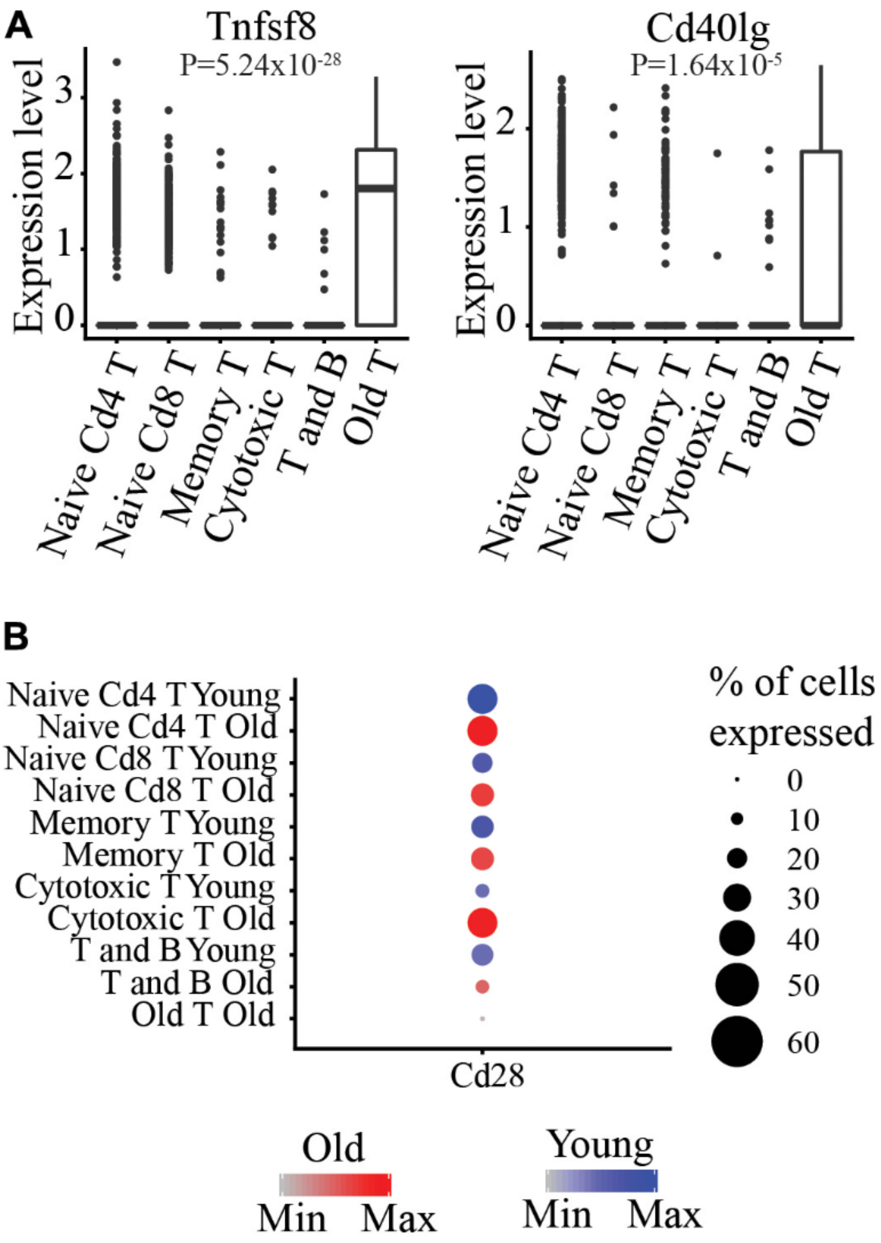

Supplementary Figure 2. (**A**) Expression level of Tnfsf8 and Cd40lg in T cells clusters. (**B**) Expression level of Cd28 in Old T cluster and other T cell clusters.
