# Supplementary material for: Single-cell transcriptomics of peripheral blood in the aging mouse
Source: Aging (Albany NY). 2023 Jan 6;15(1):6–20. doi: 10.18632/aging.204471 (PMC9876630; doi:10.18632/aging.204471)
Supplement: Supplementary Table 1 [file aging-15-204471-s002.pdf]

SUPPLEMENTARY TABLE

Supplementary Table 1. Seurat cell-cycle scoring of each cluster.

Cluster name	Phase	Count
NK	G1	135
	G2M	83
	S	194
Naive Cd8 T	G1	338
	G2M	222
	S	374
Fcer2a B	G1	2154
	G2M	1029
	S	2378
Naive Cd4 T	G1	537
	G2M	416
	S	501
DC	G1	79
	G2M	14
	S	27
Basophil	G1	17
	G2M	12
	S	19
Old T	G1	27
	G2M	16
	S	23
Macrophage	G1	50
	S	1
Cytotoxic T	G1	59
	G2M	52
	S	89
Mk	G1	132
	G2M	37
	S	106
Mono	G1	699
	G2M	184
	S	214
Memory T	G1	112
	G2M	50
	S	101
T and B	G2M	48
	S	22
Crip1 B	G1	1298
	G2M	351
	S	1085
RBC	G1	139
	G2M	73
	S	129

	**G1**	231
**Vpreb3 B**	**G2M**	93
	**S**	260
	**G1**	242
**Zcwpw1 B**	**G2M**	17
	**S**	119
